# Clinical efficacy analysis of partial cystectomy and radical cystectomy in the treatment of muscle-invasive sarcomatoid carcinoma of the urinary bladder

**DOI:** 10.3389/fonc.2024.1324487

**Published:** 2024-02-02

**Authors:** Jiansheng Xiao, Hua Chen, Jiaqi Ge, Tairong Liu

**Affiliations:** Department of Urology Surgery, Ganzhou People’s Hospital, Jiangxi Medical College, Nanchang University, Ganzhou, Jiangxi, China

**Keywords:** bladder carcinoma, carcinosarcoma, radical cystectomy, partial cystectomy, treatment

## Abstract

**Objective:**

This study compares the clinical efficacy of partial cystectomy (PC) versus radical cystectomy (RC) in the treatment of muscle-invasive bladder urothelial carcinoma (SCUB) through a retrospective analysis.

**Methods:**

We retrospectively analyzed the clinical data of 20 patients diagnosed with muscle-invasive SCUB from July 2015 to August 2023 at Ganzhou People’s Hospital. All patients underwent surgical treatment followed by chemotherapy, with 9 receiving PC and 11 undergoing RC. We compared the average survival time of deceased patients for both treatments and conducted survival and multivariate analyses using the Kaplan-Meier method and Cox proportional hazards model, respectively.

**Results:**

All 20 patients were postoperatively diagnosed with muscle-invasive SCUB and were followed up for 4 to 60 months. The average survival time for patients undergoing PC was 11.5 months, with survival rates at 1 year, 2 years, and 5 years of 55.56%, 22.22%, and 11.11%, respectively. In contrast, patients receiving RC had an extended average survival time of 22.5 months, and their 1-year, 2-year, and 5-year survival rates increased to 63.64%, 36.36%, and 18.18%, respectively. Survival analysis revealed statistically significant differences in prognosis between PC and RC for the treatment of muscle-invasive SCUB (P<0.05).

**Conclusion:**

SCUB is a rare malignant tumor with unique biological characteristics often associated with poor prognosis. Upon diagnosis, RC should be considered as an early treatment approach when the patient’s overall condition permits.

## 1 Introduction

Sarcomatoid carcinoma (SC) is a biphasic tumor composed of malignant epithelial and stromal cells, characterized by high malignancy and the potential to affect multiple organs (1–3). Current theories include the collision concept by Gorstein et al. (4) which posits that epithelial and stromal components form independently, resulting in carcinosarcoma. Another hypothesis suggests that epithelial carcinoma might transition to a stromal phenotype under the influence of the stromal microenvironment (5). Monoclonal theory proposed by Thompson et al., based on the monoclonal origin of SCUB, supports the pluripotent stem cell differentiation hypothesis and indicates clonal consistency in the tumor components (6).

SCUB has an incidence rate of 0.3% among bladder malignancies, and is considered a rare and highly aggressive tumor with a propensity for invasion and distant metastasis. Due to its rarity and distinct biological characteristics, while surgery is the treatment of choice, the selection of surgical strategies remains controversial, and there is no international consensus on treatment (7–10). According to NCCN Guidelines (11), for patients with ≤cT2 staging, a single lesion, suitable for local resection, and with sufficient negative margins, either PC or RC can be selected for treatment. This study employs retrospective analysis to investigate the clinical data of 20 patients diagnosed with SCUB at Ganzhou People’s Hospital from July 2015 to August 2023, and, with reference to pertinent literature, explores the clinical outcomes of partial and radical cystectomy in the treatment of muscle-invasive SCUB.

## 2 Materials and methods

### 2.1 Study population

This study was approved by the Ethics Committee of Ganzhou People’s Hospital, and informed consent was obtained from all patients, who each signed a consent form. The subjects of this study are 20 patients treated at Ganzhou People’s Hospital from June 2015 to August 2023, diagnosed with SCUB through cystoscopy and histopathology. Cases were excluded if patients had severe cardiac, pulmonary, or cerebrovascular diseases, were in poor overall condition for surgery, or refused surgical treatment

### 2.2 Treatment

All patients underwent preoperative urinary system CT, cystoscopy, and routine blood biochemistry examinations, which, combined with medical history, were used to assess patients’ overall condition and surgical tolerance. According to NCCN Guidelines (11), for patients with ≤cT2 staging, a single lesion, suitable for local resection, and with sufficient negative margins, either PC or RC can be selected for treatment. In this study, 11 patients received RC treatment, with 4 undergoing RC combined with ileal conduit urinary diversion, and 7 with RC followed by ureterocutaneostomy and six cycles of adjuvant chemotherapy with gemcitabine or cisplatin postoperatively. Additionally, 9 patients underwent PC via laparoscopic bladder partial resection, followed immediately by intravesical thiotepa instillation therapy: weekly for the first two months, then monthly for one year.

The study conducted regular follow-ups with patients through outpatient reviews and telephone consultations until August 2023.Patients underwent cystoscopic or full abdominal CT examinations every 3 to 6 months, with a follow-up period ranging from 4 to 60 months, until death or the end of the follow-up period.

### 2.3 Statistical analysis

In descriptive statistics, continuous variables were expressed as means ± standard deviations, while categorical variables were analyzed using the chi-square test. Survival time was calculated using the Kaplan-Meier method, with median survival times presented as medians along with 95% confidence intervals. The log-rank method was used to compare different groups, and the Cox proportional hazards model was employed for multivariate analysis to assess the independence of prognostic factors. when the P value was <0.05, comparative differences were considered statistically significant.

## 3 Results

### 3.1 Patient and tumor characteristics

**Figure 1:**
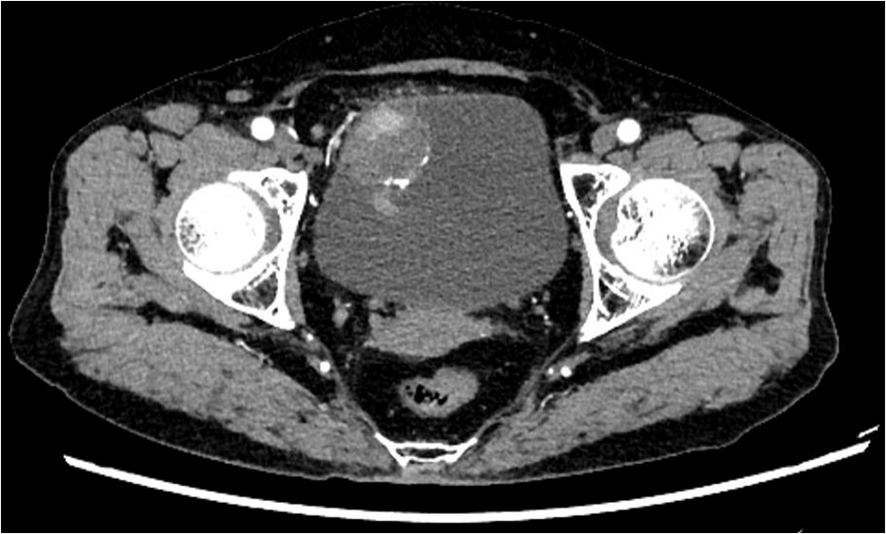
CT Abdominal Scan: Present within the bladder is a quasi-circular soft tissue mass of low density. Observed in the enhanced scan is inconsistent enhancement, and the demarcation with the anterior wall of the bladder remains obscure.

**Figure 2:**
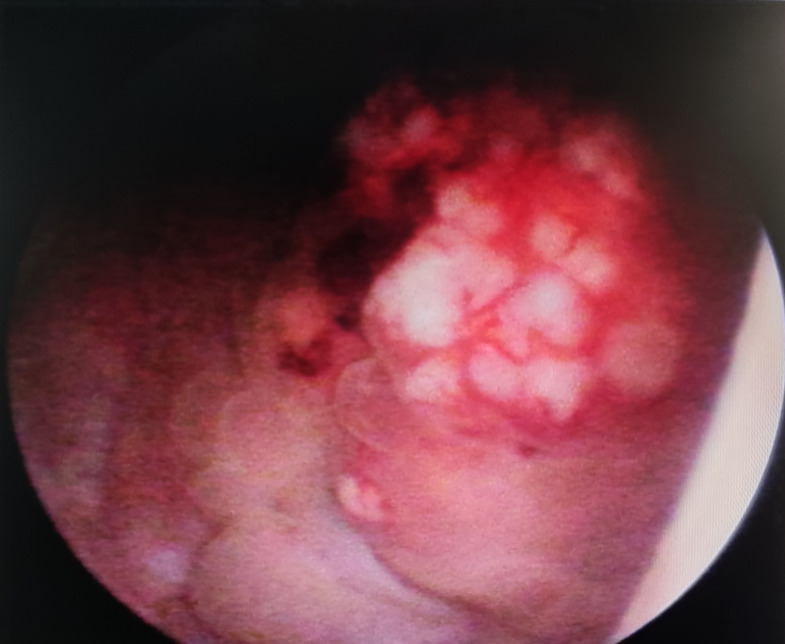
Cystoscopy: Approximately 4cm proximal to the right ureteral orifice, an elevated lesion is noticed, measuring 1cm in height and an approximate diameter of 4.1cm x 4.2cm. The lesion exterior is eroded and demonstrates easy bleeding upon contact.

The study included 20 SCUB patients, comprising 13 males and 7 females. The age at onset ranged from 44 to 82 years, with a mean age of 68.85 years, and 13 patients had a history of smoking. Lesions occurred on various bladder walls, with the lateral and posterior walls being the most common sites. The maximum diameter of the tumor specimens ranged from 2.17 to 5.01 cm, with an average diameter of 3.61cm. The postoperative pathological TNM staging was T2N0M0. Upon admission, the main complaint was gross hematuria, with some patients also presenting with urinary tract irritation symptoms. Most patients presented with gross hematuria upon admission, with some also reporting symptoms of urinary irritation. After admission, patients underwent CT scans of the urinary system, both unenhanced and enhanced, as well as cystoscopic examination. CT imaging revealed irregular soft tissue masses with uneven enhancement and no lymph node metastases detected (**Figure 1**). Cystoscopy indicated lesions on all walls of the bladder, primarily concentrated on the lateral and posterior walls. The lesions resembled cauliflower-like protrusions with a broad base; some surfaces presented with bleeding and necrosis (**Figure 2**). Immunohistochemical staining was performed on all 20 patients, with positive Ki-67 results ranging from 10% to 90% expression. Specifically, cytokeratin (CK) was positive in 18 patients, CK7 in 10 patients, epithelial membrane antigen (EMA) in 9, smooth muscle actin (SMA) in 7, P63 in 6, and GATA-3 in 4 patients. After postoperative endoscopic examination, tumor tissue showed patchy and solid distribution. Among these 20 SCUB patients, 11 underwent RC, while 9 received PC. For clinical characteristics and detailed tumor information of the two patient groups (**Table 1**).

**Table 1:** Patient and Tumor Characteristics.

Characteristics	Group	Overall N=20	PC N=9	RC N=11
Age Mean ± SD		68.85 ± 11.02	73.78 ± 6.20	64.82 ± 12.66
Gender	Male	13	7	6
	Female	7	4	3
Chronic smoking	Yes	11	6	5
	No	9	5	4
Tumor location	Trigone of bladder	2	1	1
	Anterior dome	1	1	0
	Lateral wall	9	3	6
	Posterior wall	5	3	2
	Anterior wall	3	1	2
Tumor size		3.61 ± 0.64	3.52 ± 0.36	3.67 ± 0.81
Postoperative chemotherapy		20	9	11
Immunohistochemistry	Vimentin	7	9	11
	Ki-67	10	9	11
	CK	20	8	10
	CK7	20	4	6
	EMA	18	3	6
	SMA	10	2	5
	P63	9	2	4
	GATA-3	7	1	3

### 3.2 Survival

**Figure 3:**
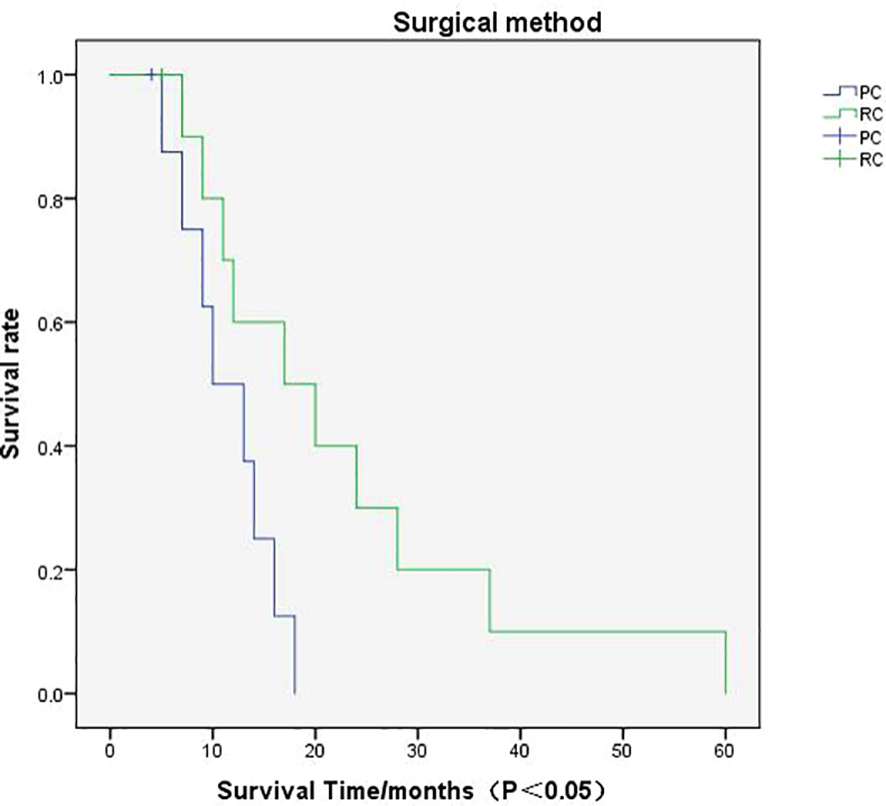
Survival Curve Analysis: The graph pertains to the survival rate associated with partial cystectomy and radical cystectomy.

Cases based solely on autopsy or death certificates and those with multiple primary tumors were excluded from the SCUB-specific survival analysis. The average follow-up period for patients in this study was 27 months (range 17 to 38 months), with a median follow-up time of 14 months (range 0 to 33 months).The 1-year survival rate was 65.00%, the 2-year survival rate was 40.00%, and the 5-year survival rate was 15.00%.Patients with SCUB who received PC treatment had an average follow-up of 12 months (range 8 to 15 months), with a median follow-up of 10 months (range 4 to 16 months), whereas those who underwent RC had an average follow-up of 23 months (range 12 to 33 months), with a median follow-up of 17 months (range 7 to 29 months), with statistically significant differences between the groups (P<0.05).Survival times for the two patient groups were analyzed using the Kaplan-Meier method (**Figure 3**), and the results showed statistically significant differences (P<0.05). The 1-year, 2-year, and 5-year survival rates for SCUB patients who received PC were 55.56%, 22.22%, and 11.11%, respectively, while for those who received RC, the corresponding survival rates were 63.64%, 36.36%, and 18.18% (**Table 2**).

**Table 2:** Median, 1-year, 2-year, and 5-year cancer specific survival of patients with SCUB according to surgical methods.

Characteristics	Survival time^*^/months (95 CI)		P-value	Survival rate (%)		
	Average survival time	Median survival time		1-year	2-year	5-year
All	27 (17~38)	14 (0~33)		65.00	40.00	15.00
PC	12 (8~15)	10 (4~16)	<0.05	55.56	22.22	11.11
RC	23 (12~33)	17 (7~29)		63.64	36.36	18.18

*: If the survival analysis time has been detected, the estimation will be limited to the maximum survival analysis time.

In the multivariable survival analysis using the Cox proportional hazards model, age, gender, tumor size, postoperative chemotherapy, and surgical approach did not emerge as independent predictors of cancer-specific survival, with only the choice of surgical method being identified as an independent variable associated with survival. Among patients with muscle-invasive SCUB, those who received PC treatment had a 5.42-fold increased risk of death compared to those receiving RC treatment (**Table 3**).

**Table 3:** COX proportional multivariate analysis of factors associated with muscle-invasive SCUB specific mortality.

Characteristics	Group	Hazard Ratio	95% CI	P value
Age	Continuous	1.67	0.32-8.66	0.538
Gender	Male	1.00	0.20-3.49	0.306
	Female	0.256		
Tumor size	Continuous	1.086	0.315-3.75	0.896
Chronic smoking	Yes	2.595	0.259-26.00	0.417
	No	1.00		
Surgical method	PC	5.42	1.14-25.83	0.034
	RC	1.00		

## 4 Discussion

SCUB is an extremely rare subtype of bladder cancer characterized by bidirectional differentiation of epithelial and mesenchymal cells (2, 12). The epithelial component can manifest in various forms, such as urothelial carcinoma, squamous cell carcinoma, small cell carcinoma, and adenocarcinoma, whereas the sarcomatoid component can exhibit a range of heterogeneity, including osteosarcoma, chondrosarcoma, and rhabdomyosarcoma (13). Given the unclear pathogenesis and low incidence of SCUB, research has primarily focused on retrospective case reports or small series (14–18), lacking prospective, multicenter, and large-sample studies. Consequently, specific treatment guidelines and uniform treatment standards have yet to be established.

RC is the standard treatment for non-metastatic muscle-invasive bladder cancer or high-risk non-muscle-invasive bladder cancer (19), despite potential complications such as infection, bleeding, thrombosis, pulmonary issues, intestinal dysfunction, and urinary tract infections that can impair quality of life (20), yet it is beneficial in preventing the spread and metastasis of cancer cells. Bladder-sparing treatments for bladder cancer have gained research attention in recent years (21, 22), PC being considered a treatment option for preserving bladder function, according to the NCCN guidelines (11). Compared to RC, the advantage of PC lies in the preservation of a part of the bladder tissue, thus maintaining bladder storage and micturition functions, allowing patients to urinate naturally through normal pathways and significantly improving the quality of life. Additionally, PC may reduce the risk of postoperative complications such as urinary tract infection and intestinal dysfunction. Furthermore, due to a smaller resection margin and less organ reconstruction required, PC typically involves shorter surgical and hospitalization durations, thereby reducing recovery and adaptation periods. However, PC also carries certain risks, potentially leaving behind cancer cells and increasing the possibility of postoperative recurrence. This study specifically targeted SCUB patients with TNM staging of T2N0M0, on whom PC and RC surgeries were respectively performed. Patients who underwent PC received regular thiotepa bladder instillations postoperatively, while those who underwent RC were treated with adjuvant gemcitabine or cisplatin to compare the clinical efficacy of the two surgical modalities.

When conducting multivariate survival analysis on SCUB patients with TNM staging T2N0M0 using the Cox proportional hazards model, this study controlled for factors such as age, gender, tumor size, and postoperative chemotherapy, finding that only the type of surgery performed was significantly associated with cancer-specific survival. Moreover, we found that the 1-year, 2-year, and 5-year survival rates of the RC group were higher than those of the PC group, with survival curve analysis indicating this difference to be statistically significant.

Based on these results, an analysis was conducted on the reasons why SCUB patients should receive RC treatment as early as possible. SCUB is a highly invasive urological malignancy, prone to infiltration and distant metastasis, with a poor prognosis (9, 12, 23). Malignant tumors of the bladder are associated with poor prognosis due to their high heterogeneity and invasiveness (24), and these characteristics are even more pronounced in SCUB, making the prognosis especially concerning (25). The high invasiveness of SCUB often leads to muscle layer invasion in most patients at initial diagnosis (8, 12), therefore, this study selected SCUB patients in the T2N0M0 stage for PC and RC surgeries to draw relevant conclusions. Prognosis associated with SCUB, early literature has reported that the staging of the tumor is a significant prognostic indicator (8). In SCUB, the sarcomatoid components are also correlated with prognosis (26), and studies have shown that sarcomatoid components with myxoid differentiation or chordoid differentiation are associated with a median survival of less than six months (17). Some research indicates that the sarcomatoid component of SC may not be directly related to prognosis (27, 28), thus more studies are needed to confirm the specific impact of sarcomatoid components on prognosis. Secondly, SCUB patients undergoing PC face a certain risk of residual cancer cells, which may lead to postoperative recurrence; conversely, RC surgery helps prevent further spread and metastasis of cancer cells. RC is the standard treatment for muscle-invasive bladder cancer (19), and although it impacts quality of life, it can significantly extend patient survival. Consistent with previous literature, the study suggests that SCUB patients should undergo RC as early as possible (13, 29). Although tumor TNM staging is an important factor in predicting cancer-specific survival (8, 10), this study selected patients all confirmed as T2N0M0 post-pathology to control for this variable. Due to the high rates of local recurrence and metastasis following surgery for SCUB, it is recommended to undergo systemic chemotherapy or intravesical chemotherapy postoperatively. Chemotherapy has the potential to eradicate microscopically residual disease and enhance the likelihood of long-term cancer control, and can improve the prognosis of patients (30). Currently, no studies have demonstrated that different chemotherapy administration methods affect the recurrence of tumors in patients. The chemotherapy regimen selected in our study includes postoperative systemic chemotherapy with cisplatin/gemcitabine, and intravesical chemotherapy with epirubicin, all patients received chemotherapy postoperatively, controlling for this variable. For SCUB patients who receive PC, there is a risk of incomplete tumor resection and residual cancer cells, which may lead to tumor recurrence (31), specifically, the high invasiveness of sarcomatoid carcinoma of the bladder can significantly reduce patient survival after recurrence. A study involving 221 patients with sarcomatoid carcinoma of the bladder found that radical cystectomy has a significant advantage in treating SCUB (8). Another study also showed that patients who underwent RC had a significantly prolonged survival (32).

Current research shows that the incidence and prevalence of SCUB are gradually increasing (33), nonetheless, research into the treatment of SCUB needs to be further deepened. This includes establishing SCUB cell line models and conducting prospective multicenter large-sample studies. Existing literature has reported the establishment of bladder cancer sarcomatoid cell line models (12): The *MaS-3 cell* line demonstrates typical characteristics of the tumor and provides information on the cell line’s genome, mutations, and proteins. This is of great significance for understanding the biology of the tumor and developing new therapies.

The limitations of this study arise from its retrospective design and a limited number of cases, which is due to the low incidence. Potential factors affecting SCUB prognosis, such as the presence of sarcomatoid components and the multiplicity of the tumor, were not adequately considered in this study. Additionally, as this research was retrospective, the patients’ prior treatment regimens consisted of cisplatin or gemcitabine therapy following radical cystectomy, and epirubicin intravesical instillation therapy following partial cystectomy. The impact of immunotherapy, targeted therapy, and other treatments on the prognosis of patients with muscle-invasive SCUB was not accounted for. After selection, this study conducted PC and RC surgeries on patients with muscle-invasive SCUB. By comparing the median survival times, 1-year, 2-year, and 5-year survival rates of both groups, and conducting survival curve analysis, this study drew its conclusions.

In summary, SCUB is a rare malignancy with distinct biological characteristics and a poor prognosis. Following diagnosis, patients should undergo radical cystectomy as soon as possible, provided their overall condition permits.

## Data availability statement

The raw data supporting the conclusions of this article will be made available by the authors, without undue reservation.

## Ethics statement

The studies involving humans were approved by the Ethics Committee of Ganzhou People’s Hospital. The studies were conducted in accordance with the local legislation and institutional requirements. The participants provided their written informed consent to participate in this study. Written informed consent was obtained from the individual(s) for the publication of any potentially identifiable images or data included in this article.

## Author contributions

JX: Writing – original draft. TL: Writing – review & editing. HC: Writing – original draft. JG: Writing – original draft.
